# Supplementary material for: Influence of conservation tillage on Greenhouse gas fluxes and crop productivity in spring-wheat agroecosystems on the Loess Plateau of China
Source: PeerJ. 2021 Apr 12;9:e11064. doi: 10.7717/peerj.11064 (PMC8048409; doi:10.7717/peerj.11064)
Supplement: File S1 [file peerj-09-11064-s001.docx]

**Influence of conservation tillage on Greenhouse gas fluxes and crop productivity in spring-wheat agroecosystems on the Loess Plateau of China**

Abdul-Rauf Malimanga Alhassan^1,2^, , Chuangjie Yang^1^, Weiwei Ma^1^, Guang Li^1^

^1^ College of Forestry, Gansu Agricultural University, Lanzhou, Gansu province, 730070, P.R. China.

^2^ Department of Water Resources Development, University of Environment and Sustainable Development, Somanya, Eastern Region, Ghana.

**EQUATIONS**

$$F=\frac{dC}{dt}\cdot\frac{M}{V_{0}}\cdot\frac{P}{P_{0}}\cdot\frac{T_{0}}{T}\cdot H (1)$$

where dC/dt is the rate of change of gas concentration; M is the molar mass of Carbon or Nitrogen (12 for CO_2_ and CH_4_ and 28 for N_2_O); V_o_ is standard molar volume of air (22.41 mol^-1^), P is the air pressure of the sampling site; P_o_ is the standard air pressure, T is the air temperature in the chamber at the sampling time, T_o_ is the standard air temperature; and H is the chamber height.

Cumulative Flux was estimated using the formula below.

$Cumulative Flux=\sum_{i=1}^{n} \left( \frac{F_{i}+F_{i+1}}{2} \right)\times24\times\left( D_{i+1}-D_{i} \right)x {10}^{-2} \ldots\ldots\ldots\ldots\ldots\ldots\ldots\ldots\ldots\ldots\ldots\ldots($2)

F_i_ and F_i+1_ denote ecosystem respiration, N_2_O and CH_4_ fluxes for previous and current day (in mg m^-2^ h^-1^) respectively; D_i_ and D_i+1_ are previous and current sampling days, respectively.

**Calculation of Carbon flux components**

Net primary productivity (NPP) was estimated as described in equation 3-7 by Bolinder et al (2007), while Gross primary production (GPP) was estimated from NPP using a factor of 0.54 as the ratio of NPP:GPP in cultivated and managed lands (Zhang et al. 2009). Grain yield and harvest index (HI) were used for calculation of NPP and GPP. It is worth noting that the CO_2_ measured by the opaque chamber is ecosystem respiration since plant community was not exposed to light and also undisturbed. From this, net CO_2_-C flux was calculated by equation 8.

$NPP=C_{P}+C_{S}+C_{R}+C_{E}$……………………………………………………………………...(3)

$C_{P}= Y_{P} \times0.45$………………………………………………………………………………..(4)

$C_{s}=\frac{Y_{P} (1-HI)}{HI} \times0.45$…………………………………………………………………………...(5)

$C_{R}=\frac{Y_{P}}{S:R\times HI}\times0.45$………………………………………………………………………………(6)

$C_{E}=C_{R} \times0.65$…………………………………………………………………………………(7)

Where

$C_{P}$is the carbon in the harvested product (grain)

$C_{s}$ is the carbon in straw

$C_{R}$ is the carbon in root tissues

$C_{E}$ is the carbon in extra root materials such as root exudate

$$Y_{P} is the grain yield, S:R is the shoot-root ratio$$

As indicated by Bolinder et al. (2007), we assumed carbon concentration in all plant parts of 0.45 kgkg^-1^ while using actual harvest indexes in our study to calculate allocations in straw and root. S:R of 9 for spring wheat was used in this study following Huang et al. (2007).

$\mathrm{Net}\mathrm{CO}_{2} flux=-GPP+R_{\mathrm{eco}}+C_{harvested crop}$………………………………………………...(8)

GPP- gross primary production, R_eco_- cumulative ecosystem respiration, C _harvested crop_- Carbon contents in harvested crops (straw and grain).

**Note: The sign convention adopted is positive (+) means emission whilst negative (-) means absorption.**

**Calculation of Global warming potential (GWP) and Greenhouse Gas Intensity (GHGI)**

Net Global warming potential (GWP) in t CO_2_ eq ha^-1^ was determined using equation 9 and Greenhouse gas intensity (GHGI) was determined following equation 10. The IPCC (2013) emission factors of 1, 34 and 298 for CO_2_, CH_4_ and N_2_O respectively were used to convert all gases to CO_2_ equivalents (CO_2_eq).

$Net GWP= \mathrm{CH}_{4}flux \times34 +N_{2}O flux\times298+Net {CO}_{2} flux\ldots\ldots\ldots\ldots\ldots\ldots\ldots\ldots\ldots\ldots..$(9)

$GHGI=\frac{GWP}{Grain yield}\ldots\ldots\ldots\ldots\ldots\ldots\ldots\ldots\ldots\ldots\ldots\ldots\ldots\ldots\ldots\ldots\ldots\ldots\ldots\ldots\ldots\ldots\ldots\ldots\ldots\ldots\ldots\ldots\ldots\ldots\ldots$(10)

The sign convention adopted is positive (+) means emission whilst negative (-) means absorption.

**Statistical equations**

$R=\alpha\times e^{\beta T}$…………………………………………………………………………………...(11)

$R=\alpha\times W^{\beta}$…………………………………………………………………………………...(12)
