# Supplementary material for: Influence of conservation tillage on Greenhouse gas fluxes and crop productivity in spring-wheat agroecosystems on the Loess Plateau of China
Source: PeerJ. 2021 Apr 12;9:e11064. doi: 10.7717/peerj.11064 (PMC8048409; doi:10.7717/peerj.11064)
Supplement: File S2 [file peerj-09-11064-s002.docx]

**Supplemental file 2: Harvest Index and Carbon Components**

**Harvest index of spring wheat under different tillage treatments in 2017-2018**

| **Treatment** | **Harvest Index (%)** |  |  |
| --- | --- | --- | --- |
|  | **2017** | **2018** | **2017-2018** |
| T | 16.82±3.02 | 18.10±5.37 | 17.41±2.92 |
| TS | 16.78±6.64 | 28.19±4.88 | 22.67±6.08 |
| NT | 19.84±8.71 | 24.89±10.19 | 22.23±9.82 |
| NTS | 21.62±7.50 | 27.52±3.20 | 24.09±5.59 |

**Carbon in plant product under different tillage treatments in 2017-2018**

| **Treatment** | **Carbon in plant product (C_P_) (kg C ha ^-1^y^-1^)** | | |
| --- | --- | --- | --- |
|  | **2017** | **2018** | **2017-2018** |
| T | 261.65±57.59 | 318.50±75.21 | 290.08±52.38 |
| TS | 264.46±28.03 | 504.55±42.24 | 384.51±34.43 |
| NT | 294.01±21.24 | 335.36±104.77 | 314.68±60.08 |
| NTS | 384.51±19.74 | 432.85±16.85 | 408.68±17.39 |

**Carbon in straw under different tillage treatments in 2017-2018**

| **Treatment** | **Carbon in straw and all aboveground residue minus grain (C_S_) (kg C ha ^-1^y^-1^)** | | |
| --- | --- | --- | --- |
|  | **2017** | **2018** | **2017-2018** |
| T | 1286.96±81.53 | 1481.71±309.25 | 1384.34±188.16 |
| TS | 1433.04±490.23 | 1320.81±335.35 | 1376.92±401.40 |
| NT | 1343.67±543.59 | 1049.78±277.52 | 1196.73±390.52 |
| NTS | 1527.14±607.61 | 1149.44±138.60 | 1338.29±344.25 |

**Gross Primary Production**

| **Period** | **GPP (kg C ha^-1^y^-1^)** | | | |
| --- | --- | --- | --- | --- |
|  | **CT** | **CTS** | **NT** | **NTS** |
| **2017** | 3563.98±314.85^a^ | 3639.55±554.05^a^ | 3656.07±714.90^a^ | 4035.73±641.31^a^ |
| **2018** | 3962.97±377.79^a^ | 4526.87±813.05^a^ | 3226.15±379.03^a^ | 3586.48±62.26^a^ |
| **2017-2018** | 3763.48±341.66^a^ | 4083.21±668.61^a^ | 3441.11±509.68^a^ | 3811.10±341.38^a^ |

**Net Primary production**

| **Period** | **NPP (kg C ha^-1^y^-1^)** | | | |
| --- | --- | --- | --- | --- |
|  | **CT** | **CTS** | **NT** | **NTS** |
| **2017** | 1924.55±170.02^a^ | 1965.36±299.19^a^ | 1974.28±386.04^a^ | 2179.30±346.31^a^ |
| **2018** | 2140.01±204.01^a^ | 2444.51±439.05^a^ | 1742.12±204.67^a^ | 1936.70±33.62^a^ |
| **2017-2018** | 2032.28±184.50^a^ | 2204.93±361.05^a^ | 1858.20±275.22^a^ | 2058.00±184.34^a^ |

**Note: tables here are presented as means ± s.e.**
